# Mechanical polarity as a driver of bone regeneration: a multiscale framework linking tension, mechanotransduction and cortical apposition

**DOI:** 10.3389/fbioe.2026.1771800

**Published:** 2026-02-19

**Authors:** Anna Ewa Kuc, Jacek Kotuła, Natalia Kuc, Joanna Lis, Beata Kawala, Michał Sarul, Magdalena Sulewska

**Affiliations:** 1 Department of Dentofacial Orthopedics and Orthodontics, Wroclaw Medical University, Wroclaw, Poland; 2 Faculty of Medicine, Medical University in Bialystok, Bialystok, Poland; 3 Department of Integrated Dentistry, Wroclaw Medical University, Wroclaw, Poland; 4 Department of Periodontal and Oral Mucosa Diseases, Medical University in Bialystok, Bialystok, Poland

**Keywords:** bone remodeling, finite element modeling, mechanobiology, osteocyte mechanotransduction, strain polarity, tensile strain

## Abstract

Mechanical loading is a fundamental regulator of bone remodeling, yet most conceptual models still focus on the magnitude of strain rather than its polarity. Here we propose a unified mechanobiological framework in which tensile-strain–dominant microenvironments act as primary drivers of cortical bone apposition, whereas compression-dominant fields predispose tissues to resorption and structural thinning. We synthesize evidence from long-bone bending, distraction osteogenesis, craniofacial suture biology, osteocyte mechanotransduction, and the periodontal ligament–alveolar complex to show that tensile strain consistently correlates with angiogenic activation, osteoblast lineage recruitment, and matrix deposition. We illustrate how subtle changes in load direction and boundary conditions can invert strain polarity in cortical regions that are classically considered “at risk” under bending or transverse displacement. We integrate these mechanical observations with canonical signaling pathways to outline a multiscale law of tension-guided bone adaptation and propose testable predictions for regenerative strategies. This perspective reframes bone mechanobiology around strain polarity and provides a conceptual scaffold for designing load-based interventions that exploit tensile fields to drive cortical regeneration across skeletal sites.

## 1 Introduction

Mechanical loading is a fundamental determinant of bone structure and function. Classic frameworks such as Wolff’s law and the mechanostat have provided foundational models for interpreting how tissues adapt to mechanical environments (Frost, 2003; Currey, 2002). Yet these paradigms primarily emphasize *strain magnitude*, while overlooking a crucial biomechanical dimension: strain polarity (tension vs. compression). In this manuscript, “strain polarity” refers to whether local deformation is tensile-dominant or compressive-dominant when expressed in terms of principal strains. Operationally, tensile dominance can be represented by the maximum principal strain (ε1 > 0), while compressive dominance can be represented by the magnitude of the minimum principal strain (|ε3|, where ε3 < 0). Polarity therefore captures the sign/directionality of the local principal strain state, which is routinely available in finite element post-processing and experimental strain measurements.

Recent studies highlight that bone adaptation is influenced not only by strain magnitude but also by other loading parameters, including frequency, rate, and waveform. Even under identical average strain magnitudes, changes in these parameters can produce distinct adaptive responses, illustrating the limitations of purely magnitude-based mechanoregulation (Wang et al., 2022; Wang et al., 2025).

A growing body of high-impact research suggests that tensile-dominant microenvironments consistently induce angiogenesis and osteogenesis, whereas compression-dominant fields predispose tissues to osteoclastic remodeling and structural thinning. This polarity-driven remodeling has been observed across multiple skeletal systems:

- Long bones: convex surfaces under tension exhibit matrix formation, while concave (compressive) surfaces undergo resorption (Skerry, 2008; Robling and Turner, 2009; Turner et al., 2009)
- Distraction osteogenesis: controlled tensile force stimulates robust intramembranous ossification within the distraction gap (Ilizarov, 1989a; Aronson, 1994; Aronson et al., 2001; Yang S. et al., 2022)
- Craniofacial sutures: tensile strain regulates sutural patency and bone formation during growth and orthopedic expansion (Herring, 2008; Mao, 2002; Mao and Nah, 2004)
- Ligamented microenvironments such as the periodontal ligament (PDL): tensile strain correlates with anabolic signaling, angiogenic activation, and matrix apposition (Jiang et al., 2021; Ei et al., 2019; Feller et al., 2015; Dutra et al., 2016; Rizk et al., 2023; Chukkapalli and Lele, 2018; Bae et al., 2024)

Across these diverse contexts, tension repeatedly appears as a conserved signal for regenerative adaptation. However, this principle has never been synthesized into a unified cross-tissue mechanobiological framework.

At the cellular level, tensile strain activates a suite of high-fidelity mechanotransductive pathways, including integrin–focal adhesion kinase (FAK)–Src signaling (Geiger et al., 2009), cytoskeletal adaptation and nuclear mechanosensing (Discher et al., 2005), Wnt/β-catenin stabilization (Bonewald, 2006; Bonewald, 2002; Bonewald, 2005; Bonewald, 2017; Nicolella et al., 2008; Duan et al., 2016; Seddiqi et al., 2023; Geoghegan et al., 2019; Li et al., 2021), and hypoxia-inducible factor-1α (HIF-1α)/vascular endothelial growth factor (VEGF)-driven angiogenic coupling (Qin et al., 2022). Conversely, sustained compression elevates receptor activator of nuclear factor κB ligand (RANKL)-mediated osteoclastogenesis and matrix degradation (Teitelbaum, 2000; Boyle et al., 2003).

These insights together suggest that mechanical polarity may serve as a multiscale regulator, linking tissue-level deformation fields to molecular programs of bone formation or resorption. Yet despite these convergent observations, existing mechanobiological models remain fragmented: computational studies quantify stresses and strains, while biological studies describe downstream signaling, without a unifying principle connecting *load direction*, *microenvironmental strain polarity*, and *remodeling outcome*.

### 1.1 Aim of this work

Strain magnitude is likely necessary to trigger a mechanobiological response, but it may be insufficient to determine the direction of remodelling (Wang et al., 2022; Wang et al., 2025; Pivonka, 2018). Indeed, the adaptive response of bone to mechanical loading depends not only on peak strain magnitude but also on other parameters such as rate, frequency, and waveform, which influence cellular mechano-responses and subsequent anabolic versus catabolic outcomes (Pivonka, 2018). We hypothesise that, once a local magnitude threshold is exceeded, strain polarity provides orthogonal information that helps determine whether the dominant outcome is anabolic (cortical apposition/activation of osteogenic programmes) or catabolic (osteoclast activation/resorption), thereby explaining why similar load magnitudes can yield opposite cortical responses depending on the sign and distribution of principal strains.

In this perspective, we propose a unified, multiscale framework wherein tensile-strain–dominant microenvironments act as a primary driver of cortical bone apposition across skeletal sites, and compression-dominant fields predispose tissues to catabolic remodeling (Stewart et al., 2020; Scott et al., 2008).

By integrating evidence from long-bone bending (Robling et al., 2006), distraction osteogenesis (Ilizarov, 1989b)*,* craniofacial suture biology (Opperman, 2000)*,* osteocyte mechanosensing (Uda et al., 2017; Hughes et al., 2020), and ligamented microenvironments (Carter et al., 1998; Epari et al., 2010), complemented by strain-based finite element models of bone adaptation (Schulte et al., 2013; Christen et al., 2014; Meslier and Shefelbine, 2023), we outline a testable mechanobiological law that reframes bone adaptation around mechanical polarity rather than magnitude alone (Stewart et al., 2020; Scott et al., 2008).

This framework offers predictive value for regenerative strategies, orthopedic device design, and therapeutic loading protocols across multiple anatomical contexts (Meslier and Shefelbine, 2023).

From a device-design perspective, the framework is intended to generate qualitative, testable predictions about how changes in alignment, stability, and load direction may shift cortical surfaces toward tensile-dominant versus compressive-dominant principal strain states (Meslier and Shefelbine, 2023). This, in turn, suggests design targets for orthopaedic and dental devices (e.g., fixation constructs, expanders, or load-sharing implants) that aim to bias the surrounding cortex toward appositional environments while minimising sustained compressive, hypoxic, and inflammatory conditions associated with catabolic remodelling (Stewart et al., 2020; Scott et al., 2008; Claes et al., 1998; Boerckel et al., 2012).

In this context, strain magnitude is necessary to trigger a mechanobiological response, whereas strain polarity provides orthogonal information that helps determine the direction of remodeling once activation thresholds are exceeded. Osteocytes play a central role in integrating these mechanical cues, and dynamic loading parameters such as frequency and strain rate may modulate mechanotransductive signaling without diminishing the importance of tensile-dominant microenvironments in promoting cortical apposition under physiological loading conditions (Hughes et al., 2020; Frost, 2003; Biewener, 1992; Barnes and Pinder, 1974; Robling et al., 2000; Robling et al., 2002).

Scope note. We focus primarily on cortical and intramembranous remodelling because polarity patterns at surfaces under bending, tension, and sustained compression are conceptually clearer and are frequently reported in clinical and experimental observations (Robling and Turner, 2009; Frost, 2003). Trabecular bone experiences more complex multiaxial loading and architecture-dependent strain states; extending the polarity framework to trabecular adaptation is an important future direction but is outside the present article’s primary scope.

## 2 Evidence across skeletal systems

Although bone adaptation has traditionally been interpreted through the lens of strain magnitude, multiple independent skeletal systems demonstrate that strain polarity—tension versus compression—is a dominant regulator of remodeling directionality. Here we synthesize evidence from long bones, distraction osteogenesis, craniofacial sutures, and ligamented microenvironments to support a cross-tissue mechanobiological principle.

### 2.1 Long-bone bending: convex tension promotes apposition, concave compression drives resorption

During long-bone bending, principal strains segregate across the cortex: tensile principal strains predominate on the convex surface, whereas compressive principal strains predominate on the concave surface. Numerous studies associate tensile-dominant surfaces with appositional modelling, while compressive-dominant surfaces more often align with resorptive or catabolic remodelling responses, depending on context and duration.

Key evidence supports this polarity-dependent response. Skerry (2008) demonstrated increased osteoblastic activity and lamellar bone deposition in tensile regions. Robling and Turner (2009) identified preferential activation of integrin–FAK and Wnt/β-catenin signalling pathways on tensile surfaces. Biewener (1992) and other researchers (Barnes and Pinder, 1974; Moreno et al., 2008) reported that bone consistently remodels to reduce peak compression while maintaining tensile stability.

**FIGURE 1:**
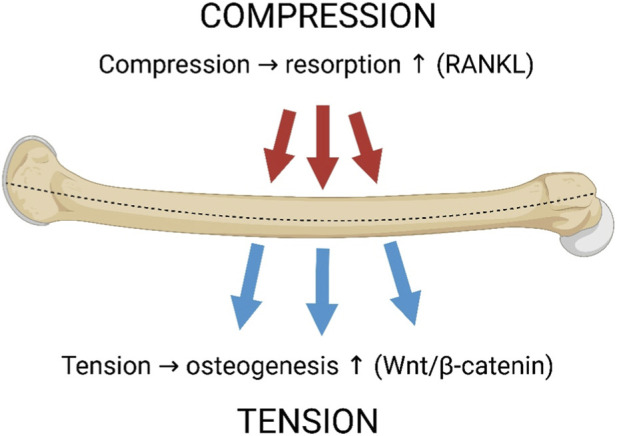
Strain polarity during long-bone bending. Schematic of a long-bone diaphysis subjected to bending. The convex surface experiences tensile strain, which promotes osteogenesis through Wnt/β-catenin signaling (blue arrows, “TENSION”), whereas the concave surface experiences compressive strain, which promotes resorption via RANKL-mediated osteoclast activation (red arrows, “COMPRESSION”). All strain fields are schematic and intended for illustration; they are not presented as outputs of new subject-specific FE simulations.

These polarity-segregated responses have been reported across multiple species and anatomical sites, suggesting that the underlying mechanobiological logic may be broadly conserved. This canonical polarity between a tensile convex surface and a compressive concave surface is illustrated schematically in Figure 1.

### 2.2 Distraction osteogenesis: tensile strain as a pure driver of *de novo* bone formation

Distraction osteogenesis (DO) provides one of the clearest biological demonstrations that controlled tensile loading alone is sufficient to generate new cortical bone. There are seminal evidence: Ilizarov (1989a) demonstrated that gradual tensile distraction induces angiogenesis, fibroblast-to-osteoblast transitions, and intramembranous ossification, Aronson (1994), Aronson et al. (2001) showed that tensile strain organizes collagen fibers along the direction of stretch and accelerates osteoblast proliferation, Claes et al. (1998), Haffner-Luntzer et al. (2016), Li et al. (2025) documented dose–response relationships between tensile magnitude and callus organization. Notably, there is no compression in the distraction gap—it is a *pure tensile environment*, and yet ossification is robust. This is powerful evidence that tension is not just permissive for bone formation—it is generative.

#### 2.2.1 Relationship to fracture-healing mechanoregulation

Concepts linking strain magnitude to tissue differentiation are well established in fracture healing and callus mechanobiology, including finite-element frameworks that relate local strain magnitude to callus formation and maturation (Lipphaus and Witzel, 2018).

The present hypothesis is complementary but distinct: we focus on cortical surface remodelling patterns and propose that the sign balance of principal strains (tensile-dominant versus compressive-dominant) helps explain why cortical thickening versus thinning can occur under comparable loading magnitudes. In other words, magnitude-based mechanoregulation may predict whether a response occurs, whereas polarity may help predict the direction of cortical remodelling once a response is engaged.

### 2.3 Craniofacial sutures: tensile strain regulates suture patency and osteogenesis

Craniofacial sutures respond exquisitely to tension by expanding, vascularizing, and depositing new bone along their edges. Key studies describe that: Herring (2008) showed that tensile loading across sutures promotes osteogenesis and maintains suture patency, Mao (2002), Mao and Nah (2004) identified tension-mediated activation of Wnt/β-catenin and osteogenic progenitors along suture borders, Opperman (2000) demonstrated that compressive loading leads to premature fusion, while tensile loading preserves growth potential. Sutural biology thus provides a natural laboratory for observing how tensile polarity maintains growth plates and drives intramembranous bone formation.

### 2.4 Ligamented microenvironments: periodontal ligament as a high-fidelity strain polarizer

Ligamented interfaces such as the periodontal ligament (PDL) amplify microstain and enable precise spatial separation of tensile and compressive responses. Evidence shows that tensile strain activates FAK- associated mechnotransduction pathways, angiogenic signaling (including VEGF), and osteogenic transcription factors (Jiang et al., 2021; Ei et al., 2019; Feller et al., 2015). Tensile-dominant regions correlate with cortical thickening, whereas compressive loading is associated with bone thinning (Dutra et al., 2016; Rizk et al., 2023; Chukkapalli and Lele, 2018; Bae et al., 2024). Importantly, the direction of loading–not merely its magnitude -dictates the local polarity of bone remodeling (Opperman, 2000; Cattaneo et al., 2009).

Clinically, we hypothesise that, under certain geometric and boundary-condition configurations, applying a traction force to a tooth may not translate into increased tensile strain at every location along the inner cortical socket wall. Subtle changes in socket geometry and load transfer could locally reduce tensile principal strain or shift the strain-polarity balance toward neutral values at specific regions. This is a testable prediction of the polarity framework and motivates targeted measurement or modelling in future work.

Although alveolar bone differs structurally from long bones or cranial sutures, it exhibits the same fundamental rule:

➡ tension → angiogenesis → osteogenesis,

➡ compression → osteoclastogenesis → thinning.

**FIGURE 2:**
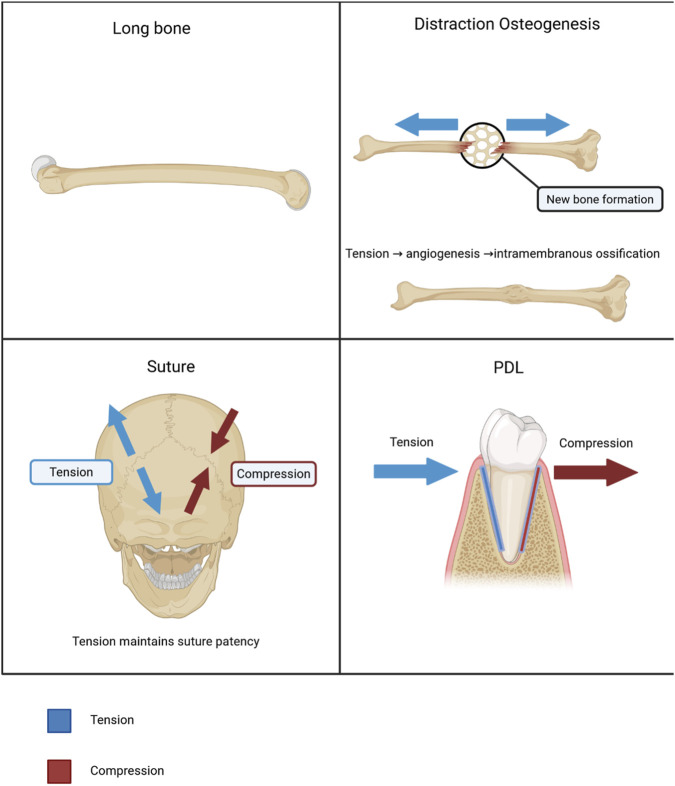
Consistent effects of strain polarity across four skeletal systems. Conceptual examples illustrating how tensile-dominated microenvironments favor bone formation, whereas compressive-dominated regions favor resorption or closure. Panels show (top left) a long bone, (top right) distraction osteogenesis, where axial tension in the regenerate drives angiogenesis and intramembranous ossification, (bottom left) cranial sutures, where tension maintains suture patency, and (bottom right) the periodontal ligament (PDL), in which tension and compression act on opposite sides of the tooth root. Blue arrows indicate tension; red arrows indicate compression.

This convergence across anatomically diverse systems suggests a shared mechanobiological principle. These cross-tissue observations are summarized in Figure 2, which compares strain polarity and remodeling outcomes across long bones, distraction osteogenesis, cranial sutures and the periodontal ligament.

## 3 Multiscale mechanical polarity framework

**FIGURE 3:**
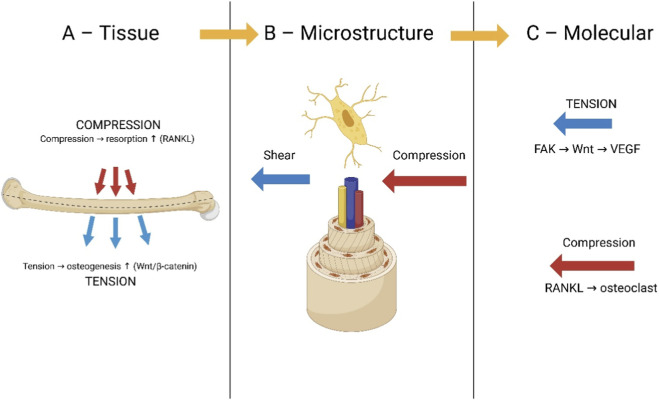
Multiscale view of strain polarity in bone mechanobiology. **(A)** Tissue level: bending of a long bone produces polarity between tensile and compressive surfaces. **(B)** Microstructural level: strain polarity and fluid shear are sensed by osteocytes within the lacuno–canalicular network. **(C)** Molecular level: tensile strain predominantly activates FAK–Wnt–VEGF signaling, favoring angiogenesis and osteogenesis, whereas compressive strain upregulates RANKL, favoring osteoclastogenesis and resorption. All strain fields are schematic and intended for illustration; they are not presented as outputs of new subject-specific FE simulations.

Bone is often modeled as a material responding to *strain magnitude*, yet experimental and computational analyses consistently demonstrate that the orientation and polarity of strain fields exert a decisive influence on remodeling direction. Here we outline a multiscale framework in which tensile-dominant microenvironments robustly activate osteogenic programs, while compression-dominant fields promote catabolic remodeling, integrating principles from tissue mechanics, fluid–structure interactions, and osteocyte biology. Our proposed multiscale framework is outlined in Figure 3, linking tissue-level strain polarity, microstructural fluid–structure interactions and polarity-specific molecular signaling cascades.

Most computational remodelling frameworks quantify the mechanical stimulus primarily through magnitude-based measures such as strain energy density, effective strain, or related scalar invariants, often combined with spatial averaging or influence functions derived from the local strain field to represent cellular mechanosensing at the tissue scale (Safira et al., 2024). These approaches have produced valuable predictions but typically treat tension and compression symmetrically unless additional assumptions are introduced.

Related work has also incorporated directionality indirectly by representing bone as anisotropic or orthotropic, thereby allowing remodelling to depend on material axes and directional stiffness (Mathai et al., 2021). While these formulations acknowledge directional effects, they do not explicitly encode a polarity rule that distinguishes tensile-dominant from compressive-dominant environments as a biological switch between apposition and resorption. This unresolved distinction motivates the present hypothesis-driven framework.

### 3.1 Tissue-level polarity: how small directional shifts invert remodeling outcomes

**FIGURE 4:**
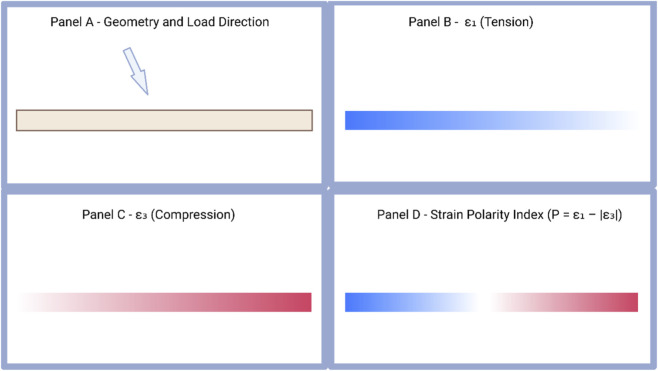
Conceptual finite element representation of strain polarity in a simplified cortical beam. **(A)** Geometry and load direction for a simplified cortical beam subjected to an oblique force. **(B)** Spatial distribution of the maximum principal tensile strain (ε_1_, tension). **(C)** Spatial distribution of the minimum principal compressive strain (ε_3_, compression). **(D)** Strain-polarity index $P=\varepsilon_{1}-|\varepsilon_{3}|$ , highlighting tensile-dominated regions (P > 0), compressive-dominated regions (P < 0), and a near-balanced transition zone. All strain fields are schematic and intended for illustration; they are not presented as outputs of new subject-specific FE simulations.

Finite element analyses of long bones, craniofacial structures, and alveolar complexes demonstrate that even small changes in load vector orientation can invert local strain polarity, transforming regions of compression into tensile-dominant microenvironments (Cattaneo et al., 2009; Jang et al., 2016). A conceptual finite element representation of this polarity in a simplified cortical beam is shown in Figure 4.

Across skeletal systems, bending generates non-uniform and dynamically redistributed tensile and compressive strain fields as a function of geometry and loading configuration (Bu and rr, 2002), a phenomenon further corroborated by both experimental and theoretical investigations into bone microdamage and mechanobiology (Carter et al., 1998; Taylor and Lee, 2003; Diab and Vashishth, 2005; Dominguez and Agnew, 2019; Donahue and Galley, 2006; Naples et al., 2001). In craniofacial sutures, even subtle asymmetric tensile loading maintains persistent osteogenic fronts, whereas compressive forces promote sutural constriction (Mao, 2002; Mao and Nah, 2004). In ligamented microenvironments such as the periodontal ligament (PDL), the direction of loading - rather than its magnitude - determines whether the ligament experiences tensile expansion or compressive collapse, thereby dictating patterns of alveolar bone remodeling (Jiang et al., 2021; Ei et al., 2019; Feller et al., 2015; Cattaneo et al., 2009; Jang et al., 2016).

These findings collectively suggest a simple principle: strain polarity, not magnitude, dictates anabolic versus catabolic bone responses.

### 3.2 Microstructural amplification: role of interfaces, anisotropy, and local heterogeneity

Although the tissue-level polarity variable is expressed here in terms of principal tension versus compression, cells often sense these environments through shear-related mechanisms, including interstitial fluid flow and membrane/cytoskeletal shear at focal adhesions (Bonewald, 2011; Weinbaum et al., 1994). Thus, shear stress and flow-related cues can serve as mechanistic intermediates through which tensile-dominant versus compressive-dominant tissue states are transduced into biochemical signalling.

Bone is not a homogeneous solid: the cortical matrix, trabeculae, and ligamented interfaces exhibit distinctive anisotropies that amplify or dampen tensile and compressive components.

Key mechanisms include: osteonal architecture channels tensile strains along collagen fiber orientation, enhancing mechanosensitive signaling (Ramasamy, 2017; Hendriks and Ramasamy, 2020), fluid–structure interactions produce shear stresses in lacuno-canalicular networks that scale directly with tensile deformation (Weinbaum et al., 1994; Yang F. et al., 2022), thin cortical shells (alveolar bone, calvarium) show dramatically amplified polarity gradients even at low overall strain (Cattaneo et al., 2009; Jang et al., 2016), ligamented tissues (PDL, sutures) act as “strain polarity amplifiers,” converting small directional loading differences into large strain differentials (Herring, 2008).

This multiscale heterogeneity further supports the concept that strain polarity propagates downward, from whole-bone mechanics to microstructural deformation patterns (Cattaneo et al., 2009; Jang et al., 2016).

At the microstructural level, the biological distinction between tensile- and compressive-dominant strain fields can be understood through their differential effects on lacuno–canalicular fluid flow. Tensile deformation tends to increase canalicular patency and generate directional pressure gradients, thereby promoting interstitial fluid flow and elevated fluid shear stress on osteocyte dendritic processes. These shear forces are well established as primary activators of osteocytic mechanotransduction, inducing calcium signaling, COX-2 and prostaglandin production, and downstream activation of Wnt/β-catenin and VEGF-mediated angiogenic pathways (Bonewald, 2011; Weinbaum et al., 1994; Kapur et al., 2003).

In contrast, compressive-dominant strain fields are more likely to reduce canalicular dimensions, dampen fluid flow, and increase hydrostatic pressure, resulting in lower shear stress and a signaling milieu that favors sclerostin expression, RANKL upregulation, and inflammatory mediator release. This mechanistic divergence provides a plausible explanation for how two regions experiencing similar strain magnitudes but opposite strain polarity can activate fundamentally different molecular programs. We therefore hypothesize that strain polarity acts upstream of molecular signaling by shaping the directionality and magnitude of lacuno–canalicular fluid flow, thereby serving as a key discriminator between anabolic and catabolic remodeling responses (Uda et al., 2017; Weinbaum et al., 1994; Delgado-Calle and Bellido, 2015).

### 3.3 Cellular mechanosensing: polarity-aware activation of bone-forming vs. bone-resorbing programs

Mechanical polarity is not merely a structural concept—osteocytes, fibroblasts, and osteoblast-lineage cells exhibit distinct molecular responses to tension vs. compression.

Tension-activated programs include integrin–FAK–Src signaling, which promotes cytoskeletal organization and Runx2 activation (Geiger et al., 2009; Geoghegan et al., 2019). Preferential stabilization of Wnt/β-catenin in tensile-dominant mechanical environments enhances osteoblast differentiation and bone formation (Bonewald, 2006; Bonewald, 2002; Bonewald, 2005; Bonewald, 2017; Duan et al., 2016). In pararell, HIF-1α/VEGF signaling couples tensile strain to angiogenic activation, a prerequisite for effective cortical apposition (Qin et al., 2022). At the subcellular level, cytoskeletal stretching induces deformation and elongation of the nuclear lamina, increasing chromatin accessibility and transcriptional competence (Discher et al., 2005; Weinbaum et al., 1994; Yang F. et al., 2022).

In contrast, compression-dominant environments are associated with increased RANKL/osteoprotegerin (OPG) signaling and osteoclastogenesis (Boyle et al., 2003), as well as induction of inflammatory cytokines such as interleukin-1β (IL-1β), tumour necrosis factor-α (TNF-α), which suppress osteoblast lineage commitment and matrix production (Teitelbaum, 2000).

Thus, tension and compression do not represent opposite magnitudes of the same mechanical signal; they activate fundamentally distinct molecular cascades that bias skeletal remodeling toward anabolic or catabolic outcomes.

### 3.4 Mapping mechanical polarity to biological outcomes: a multiscale law

The evidence across skeletal tissues and molecular pathways converges into a single, testable principle:

If tensile strain dominates within a cortical or periosteal microenvironment, below damage thresholds and above mechanotransductive activation thresholds, the net remodeling response will be bone apposition. If compressive strain dominates, the response will shift toward bone resorption or thinning.

This principle is consistent with long-bone bending adaptation, distraction osteogenesis gap healing, suture patency and expansion, ligamented microenvironments such as the PDL, and osteocyte shear-dependent activation patterns (Wang et al., 2022; Wang et al., 2025; Weinbaum et al., 1994; Yang F. et al., 2022; Gremminger and Phillips, 2021).

The implication is profound - bone remodeling is polarity-driven, not magnitude-driven.

## 4 Molecular mechanotransduction under tensile vs. compressive loading

Mechanical stimuli regulate bone remodeling through a hierarchy of molecular pathways that distinguish sharply between tensile and compressive microenvironments. Rather than representing opposite ends of a single continuum, tension and compression trigger qualitatively distinct mechanotransductive programs, leading respectively to osteogenesis or osteoclastogenic remodeling. Here, we synthesize evidence from high-impact studies on osteocytes, periosteal progenitors, fibroblastic tissues, and intramembranous bone to outline a polarity-aware mechanobiological framework.

### 4.1 Tension activates anabolic pathways: integrin–FAK–Src, Wnt/β-catenin, and angiogenic coupling

Tensile deformation directly activates the integrin–FAK–Src axis, leading to cytoskeletal tension, focal adhesion maturation, and nuclear mechanosensing.

Geiger et al. (2009) demonstrated that tensile deformation strengthens focal adhesion complexes and increases FAK phosphorylation, coordinating downstream osteogenic signaling (Geiger et al., 2009). Discher et al., (2005) and Zhang et al. (2021) showed that mechanical stretching induces nuclear flattening and increased chromatin accessibility, thereby promoting osteogenic gene programs and biasing mesenchymal stem cells toward an osteogenic lineage.

A crucial downstream consequence is β-catenin stabilization, which serves as a central node linking tensile strain to osteoblast differentiation via Wnt signaling (Krishnan et al., 2006; Yan et al., 2016). Bonewald identified osteocyte-mediated Wnt/β-catenin activation as a hallmark of tensile mechanotransduction (Bonewald, 2006; Bonewald, 2002; Bonewald, 2005; Bonewald, 2017). Robling and Turner (2009) reported that Wnt signaling is upregulated specifically on tensile surfaces of long bones during mechanical loading (Robling and Turner, 2009). Tension also couples to angiogenesis, which is required for cortical apposition–Qin et al. (2022) and Ramasamy et al. (2014, 2016) demonstrated that mechanical stimulation upregulates HIF-1α/VEGF expression and coordinates vascular ingrowth with osteoblast recruitment (Qin et al., 2022; Ramasamy et al., 2014; Ramasamy et al., 2016). Collectively, these findings show that tensile strain converges on an angiogenic–osteogenic axis, enabling bone formation across skeletal tissues.

### 4.2 Compression triggers catabolic remodeling: RANKL/OPG imbalance and inflammatory cytokine activation

In contrast, compressive loading—particularly hydrostatic pressure and matrix compaction—activates osteoclastogenic pathways, thereby favoring bone resorption over formation. There are key molecular signatures of compression. Boyle et al. (2003) demonstrated that compression elevates RANKL expression, tipping the RANKL/OPG ratio toward osteoclast differentiation (Boyle et al., 2003). Teitelbaum (2000) established that compressive and inflammatory stimuli synergistically amplify osteoclastogenesis (Teitelbaum, 2000). Compressive strain induces IL-1β, TNF-α, and PGE_2_ in fibroblastic and osteoblastic populations, thereby shifting the RANKL/OPG balance toward osteoclastogenic signaling and suppressing osteogenic lineage commitment (Seddiqi et al., 2023; Teitelbaum, 2000; Kanzaki et al., 2002).

Furthermore, osteocytes within compressed bone experience altered interstitial fluid flow in the lacuno-canalicular network, leading to changes in fluid shear stress on their processes and modulating mechanosensory signaling. This reduction in fluid shear under compressive conditions correlates with suppressed anabolic signaling, including decreased COX-2/prostaglandin activity, and can impair osteoblast recruitment (Weinbaum et al., 1994; Yang F. et al., 2022; Kapur et al., 2003; Norvell et al., 1985). Weinbaum’s lacuno-canalicular model supports that fluid shear—rather than static hydrostatic pressure—is the primary activator of osteocytic mechanosensing, explaining why compression often corresponds to local biological quiescence despite the activation of osteoclastogenic pathways (Weinbaum et al., 1994). These observations highlight the central role of osteocytes as mechanosensors that integrate compressive signals and modulate downstream bone remodeling.

### 4.3 Osteocytes as polarity discriminators: the central mechanosensory hypothesis

Osteocytes represent the primary mechanosensors of bone. Their lacuno-canalicular network is exquisitely sensitive to the directionality of deformation, not merely its magnitude, allowing the bone to distinguish between tensile and compressive strains. Mechanisms supporting polarity detection include: tension increasing canalicular fluid shear and stretch-induced Ca^2+^ oscillations, which promote anabolic gene expression (Bonewald, 2002; Bonewald, 2011); compression decreasing canalicular shear while increasing hydrostatic pressure, shifting signaling toward sclerostin production and osteoclastogenic cues via antagonism of Wnt/β-catenin pathways (Jia et al., 2020; Lin et al., 2009; Delgado-Calle and Bellido, 2022); and cytoskeletal “tensegrity” models proposing that tensile strain enhances nuclear mechanotransduction via LINC complexes and integrin-mediated focal adhesion signaling (Discher et al., 2005; Ingber, 2008). Downstream signaling from osteocytes can further influence osteoblast differentiation via YAP/TAZ-mediated pathways (Xiong et al., 2018). Thus, osteocytes act as polarity-sensitive transducers, biasing bone toward formation or resorption depending on the vector orientation of applied loads.

### 4.4 From mechanical fields to cellular response: an integrated polarity map

Across skeletal tissues, the evidence converges on a single coherence:

✔ Tension → shear → FAK → Wnt → VEGF → osteogenesis.

✔ Compression → hydrostatic pressure → RANKL → inflammatory cytokines → osteoclastogenesis.

This mapping is conserved across long bones, craniofacial sutures, distraction osteogenesis, and ligamented environments—despite vast differences in structure and embryologic origin. Such cross-tissue coherence strongly supports the hypothesis that mechanical polarity, rather than magnitude, is the principal organizer of bone remodeling programs.

While our polarity-based mapping captures the dominant mechanobiological trends, exceptions may arise under dynamic compression, high-frequency loading, or microarchitectural configurations that generate shear despite net compressive strain. These factors should be considered when interpreting polarity in tissues with complex geometry or mixed loading patterns.

At the tissue scale, tensile-dominant principal strain fields can promote matrix–cell relative motion and focal adhesion loading. This mechanical cue is sensed as shear or traction at integrin complexes, activating focal adhesion kinase (FAK)/Src pathways and pro-osteogenic signaling, including Wnt/β-catenin and VEGF pathways (Wang et al., 2022; Krishnan et al., 2006). In contrast, sustained compressive-dominant environments can reduce perfusion, increase hypoxia-responsive signals (e.g., HIF-1α) and inflammatory signals (e.g., IL-1β, TNF-α), and shift remodeling toward RANKL expression and osteoclast activation (Boyle et al., 2003). Importantly, the effects of mechanical signals on bone adaptation depend not only on magnitude but also on parameters such as frequency, strain rate, and waveform, which may account for exceptions under dynamic or complex loading patterns (Wang et al., 2025).

## 5 Proposed Strain Polarity Law and testable predictions

The convergence of evidence across long bones, distraction osteogenesis, craniofacial sutures, and ligamented microenvironments suggests that tensile strain may act as the principal mechanobiological determinant of cortical bone apposition, independent of anatomical location, embryologic origin, or loading modality.

We therefore propose the following generalizable principle:

### 5.1 Strain Polarity Law

If tensile strain dominates over compressive strain in a given cortical or periosteal microenvironment, and both remain within physiological (non-damaging) ranges, the net remodeling response will favor osteogenic matrix apposition. Conversely, if compressive strain dominates, the net remodeling response will shift toward bone resorption, thinning, or remodeling arrest.

This principle integrates:

- tissue-level phenomena (bending asymmetry, distraction osteogenesis gap formation),
- microstructural behavior (shear amplification in the lacuno-canalicular system),
- cellular mechanotransduction (integrin–FAK–Src vs. RANKL/OPG signaling),
- vascular coupling (HIF-1α/VEGF activation under tension),
- lineage dynamics (osteoblast vs. osteoclast differentiation).

Although the Strain Polarity Law is formulated broadly, its primary domain of applicability is cortical and periosteal microenvironments where directional strain fields are well defined. Trabecular bone and regions under predominantly axial compression may exhibit more complex behaviors depending on loading frequency, fluid shear, and microarchitecture. Therefore, the law should be interpreted as a framework that applies most robustly to cortical surfaces exposed to bending, tensile distraction, or ligament-mediated loading. The Law does not require exact strain *magnitudes* to be conserved across regions, but instead posits that strain polarity—the sign and vector orientation of deformation—is the primary organizing variable in skeletal adaptation.

### 5.2 Mathematical formulation

Let ε1 denote the maximum principal strain and ε3 denote the minimum principal strain (ε1 ≥ ε2 ≥ ε3). We define a tensile component as ε_tension = max(ε1, 0) and a compressive component as ε_compression = max(|ε3|, 0). A simple polarity index can then be expressed as:

- *P* = (ε_tension − ε_compression)/(ε_tension + ε_compression + ε0),

where ε0 is a small positive constant to avoid division by zero. By construction, *P* → +1 indicates tensile dominance, *P* → −1 indicates compressive dominance, and *P* ≈ 0 indicates a near-balanced or neutral state.

- T_min → lower tensile threshold for osteogenic activation.
- C_max → upper compressive threshold for catabolic activation.

Then:

If *P* > T_min → osteogenic zone.

If *P* < −C_max → resorptive zone.

If −C_max < P < T_min → quiescent zone (balanced remodeling)

Relation to the “lazy zone”. Classic remodelling theories propose a magnitude-based homeostatic window in which bone mass is relatively stable (“lazy zone”), originally described in the mechanostat framework by Carter (1984). The polarity framework is complementary: within any given magnitude regime, the sign balance of principal strains may bias the local response toward apposition versus resorption. Conceptually, a magnitude threshold may govern whether adaptation is triggered, while polarity may help determine the direction of adaptation once triggered.

This formulation mirrors the mechanostat concept but introduces directionality, something absent in classical strain-magnitude theories. It makes the law testable and allows translational use in: implant design, orthopedic load optimization, craniofacial development modeling, regenerative engineering.

**FIGURE 5:**
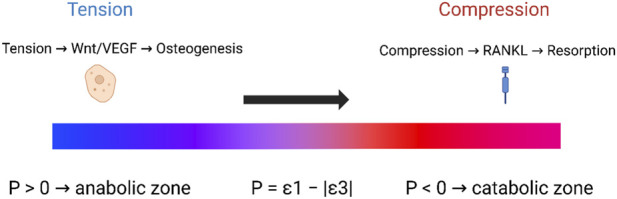
Conceptual the strain-polarity law and anabolic/catabolic domains. Conceptual diagram of the signed strain-polarity index $P=\varepsilon_{1}-|\varepsilon_{3}|$ . Positive values (*P* > 0) define a tension-dominated, anabolic zone linked to Wnt/VEGF-driven osteogenesis, whereas negative values (*P* < 0) define a compression-dominated, catabolic zone linked to RANKL-driven resorption. Values near zero correspond to a near-balanced, quiescent remodeling state along the central portion of the gradient, conceptually analogous to the physiological “lazy zone” described in classical mechanostat theory by Carter (1984).

In this formulation, ε_tension denotes the maximum principal tensile strain (ε_1_ ≥ 0), whereas ε_compression refers to the magnitude of the minimum principal compressive strain (|ε_3_|). We define a signed strain-polarity index, *P* = ε_1_ − |ε_3_|: *P* > 0 indicates tensile dominance, *P* < 0 indicates compressive dominance, and *P* ≈ 0 indicates a near-balanced state. In mixed or shear-dominated regions, *P* can be computed from principal strains obtained through finite element analysis or full-field digital image/volume correlation (DIC/DVC). The Strain Polarity Law is depicted conceptually in Figure 5, where the signed strain-polarity index $P=\varepsilon_{1}-|\varepsilon_{3}|$ spans from tensile, anabolic domains (*P* > 0) to compressive, catabolic domains (*P* < 0), with a near-balanced, quiescent zone around *P* ≈ 0.

### 5.3 Testable predictions arising from the Strain Polarity Law

Prediction 1 — Local tensile augmentation induces cortical thickening.

Increasing tensile bias (via load direction, muscle recruitment, or interface constraint) will locally thicken cortical bone even if global loading magnitude remains unchanged (Wang et al., 2025; Zhang et al., 2021; Giorgio et al., 2023).

Can be tested using: micro-CT longitudinal imaging, digital volume correlation, finite element strain maps.

Testing approach. Longitudinal imaging (e.g., CT/µCT) can quantify regional cortical thickness changes over time, while FE-style strain maps (from existing models or simplified analyses) can identify regions predicted to be tensile-dominant (*P* > 0) versus compressive-dominant (*P* < 0). The hypothesis predicts that thickness increases will spatially align with tensile-dominant regions more consistently than with high magnitude alone, particularly in cases where magnitude is comparable across opposing surfaces but polarity differs.

Prediction 2 — Compression-dominant remodeling will preferentially activate RANKL and inflammatory mediators.

Regions with *P* < −C_max will exhibit increased osteoclast markers, reduced osteoblast activity, and decreased vascular density (Branecka et al., 2022).

Testable via: histology, TRAP staining, vascular perfusion assays.

Prediction 3 — Strain polarity governs suture behavior.

Suture patency or fusion can be predicted primarily by the sign of ε, rather than its magnitude (Kopher and Mao, 2003). Small tensile preloads maintain sutural osteogenesis; small compressive preloads promote constriction.

Testable via: longitudinal imaging of sutures (micro-CT, µCT), histological analysis, and molecular markers of osteogenesis (e.g., osteocalcin, alkaline phosphatase).

Testing approach: Experimental models can apply controlled tensile or compressive preloads to sutural regions. The hypothesis predicts that regions under tensile-dominant conditions will maintain or increase osteogenic activity, while regions under compressive-dominant conditions will show reduced osteoblast activity and earlier suture closure. This allows direct testing of the Strain Polarity Law in craniofacial or other sutural tissues.

Prediction 4 — Tensile vector alignment governs distraction osteogenesis quality.

Gap mineralization will be proportional to directional coherence of tensile vectors, not total distraction force (Zhang et al., 2021; Li et al., 2020). Osteogenesis during distraction osteogenesis is influenced not only by loading magnitude but also by how tensile strain directionally organizes cellular and molecular responses.

Experimental models: rodent DO models with altered distraction trajectories.

Testable via: controlled loading/expansion protocols with regional strain estimation (e.g., DIC in accessible models or validated computational surrogates) and molecular readouts (VEGF, HIF-1α, RANKL/OPG) sampled from tensile-dominant versus compressive-dominant regions.

Prediction 5 — Ligamented microenvironments act as polarity amplifiers.

Fibrous tissues (e.g., PDL, sutures, periosteum) will magnify the tensile component and suppress compression under anisotropic loading (Ramasamy et al., 2016; Li et al., 2019).

Therefore, the law predicts enhanced osteogenic responses in these tissues, even at low global strain.

Evidence: periodontal ligament, periosteal strain studies.

Testable via: measurement of local strain in fibrous tissues using micro-CT, digital volume correlation, or strain gauges, combined with molecular readouts of osteogenesis (e.g., MAR, BFR, VEGF) and remodeling markers.

Prediction 6 — Orthopedic implants can be optimized by designing tensile-dominant periprosthetic fields.

Hip stems, tibial plates, spine cages, and maxillofacial implants that create a tensile bias are predicted to reduce stress shielding and cortical thinning (Giorgio et al., 2023; Sadighi et al., 2025; Zou et al., 2024; Munford et al., 2022).

This opens engineering pathways with huge translational impact.

For clarity, each prediction can be operationalized using (a) polarity index *P* derived from principal strains, (b) biological endpoints such as MAR/BFR, cortical thickness, TRAP activity, or VEGF expression, and (c) time scales ranging from early molecular responses (hours–days) to architectural changes (weeks–months).

These standardized metrics facilitate experimental validation across laboratories.

### 5.4 Implications for regenerative medicine and design of load-based therapies

- Tissue engineering - bioreactors should incorporate *tension-dominant regimes* for inducing osteogenic maturation of MSCs (consistent with Discher’s mechanosensing framework).
- Implant design - mechanical interfaces should promote tensile strain in peri-implant cortical zones to avoid resorption (stress shielding) and promote osseointegration.
- Growth modification–craniofacial orthopedic devices should be designed to bias sutures toward tensile opening rather than compressive constriction.
- Rehabilitative loading - physical therapy protocols should incorporate directional loading cues to stimulate bone regeneration without overloading joints.

### 5.5 Limitations and pathways for experimental validation

While the present framework emphasizes strain polarity as the primary determinant of anabolic versus catabolic bone remodeling, we acknowledge that strain magnitude, frequency, and temporal characteristics can conditionally override polarity-driven responses under specific loading regimes. In particular, high-frequency or rapidly oscillatory compressive loading, such as that generated during weight-bearing exercise, jumping, or vibration-based interventions, has been shown to induce net bone formation despite a locally compressive principal strain state. This apparent paradox is explained by the fact that dynamic compression generates large oscillatory pressure gradients within the lacuno–canalicular network, resulting in elevated interstitial fluid flow and shear stress acting on osteocyte processes, which are known to be potent anabolic stimuli (Bonewald, 2006; Turner, 1998; Weinbaum et al., 1994).

We therefore propose the existence of a magnitude- and frequency-dependent override threshold, above which dynamic compressive loading can elicit osteogenic responses through shear-mediated mechanotransduction, even in the absence of tensile-dominant principal strains. Importantly, this override does not contradict the strain polarity framework, but rather defines a conditional regime in which compressive fields converge on the same downstream shear-sensitive signaling pathways as tensile environments (Robling and Turner, 2009; Rubin et al., 2004; Rubin et al., 2001). By contrast, static or sustained compressive loading predominantly induces reduced fluid flow, hypoxia, inflammatory signaling, and osteoclastogenic remodeling (Teitelbaum, 2000).

The Strain Polarity Law is, at this stage, a conceptual, cross-tissue framework rather than a fully validated constitutive model. As such, it deliberately simplifies several important aspects of bone mechanobiology. First, we focus on the balance between tensile and compressive principal strains, whereas real bony tissues experience complex combinations of tension, compression, shear and hydrostatic loading that vary over time. In particular, dynamic compression at high frequencies, fluid shear in the lacuno–canalicular network and anisotropic collagen orientation may all modulate cell responses in ways that are not fully captured by a single scalar index (*P*).

Second, our formulation is tailored to cortical and intramembranous bone surfaces and may not directly generalize to trabecular lattices or endochondral growth plates, where architecture and mechanotransduction differ. Third, the proposed thresholds (T_{\min}) and (C_{\max}) are currently qualitative and context-dependent; they are intended as conceptual markers rather than universal numerical constants.

These limitations can be addressed by a combination of *in silico* and *in vivo* studies. Full-field digital image or volume correlation (DIC/DVC) coupled with high-resolution imaging under controlled loading could be used to map principal strains in cortical regions that are known to thicken or thin in response to altered mechanics. Multiphysics finite element models that incorporate anisotropic collagen alignment, poroelastic fluid flow and realistic boundary conditions can then be used to compute (*P*) and explore how changes in load direction, magnitude and fixation shift tissues between anabolic, quiescent and catabolic domains.

Finally, targeted animal experiments that deliberately switch local strain polarity—while keeping strain magnitude similar—would provide a direct test of whether polarity, rather than intensity alone, is the primary driver of cortical apposition versus resorption.

## 6 Conclusions and future directions

The integration of mechanical, microstructural and molecular perspectives developed in this work suggests that strain polarity is a unifying mechanobiological principle across long bones, distraction osteogenesis, craniofacial sutures and ligamented interfaces. When tensile principal strains dominate over compressive ones in a given cortical microenvironment (P > 0), osteocytes and their periosteal or endosteal neighbors preferentially activate Wnt/VEGF-linked anabolic programs and drive appositional growth. When compressive strains dominate (*P* < 0), RANKL-mediated osteoclastogenesis and cortical thinning become more likely. Near-balanced states correspond to quiescent remodeling.

The proposed Strain Polarity Law and associated index ( $P=\varepsilon_{1}-|\varepsilon_{3}|$ ) provide a compact language for describing these tendencies and for translating mechanobiological insight into engineering practice. They offer a basis for designing implants and fixation systems that bias interfaces toward tensile, osteogenic polarity; for planning distraction and craniofacial orthopedic protocols that maintain tension-dominated gaps and sutures; and for developing rehabilitation regimens that intentionally steer cortical regions into anabolic rather than catabolic domains.

Future work should focus on quantifying realistic ranges of (*P*) *in vivo*, calibrating context-specific thresholds for different bones and age groups, and embedding the polarity framework into multiscale computational models that couple loading, microarchitecture and cellular signaling. By turning a qualitative “tension versus compression” intuition into a testable, quantitative construct, the strain-polarity perspective can help bridge classical bone biology, biomechanics and biomedical engineering, and ultimately support more rational, mechanism-based strategies to stimulate cortical regeneration and prevent resorptive remodeling.

## Data Availability

The original contributions presented in the study are included in the article/supplementary material, further inquiries can be directed to the corresponding author.
